# Influence of Residue Soil on the Properties of Fly Ash–Slag-Based Geopolymer Materials for 3D Printing

**DOI:** 10.3390/ma17122992

**Published:** 2024-06-18

**Authors:** Zhijie Zhou, Jian Geng, Chen Jin, Genjin Liu, Zhenjiang Xia

**Affiliations:** 1 College of Civil Engineering and Architecture, Zhejiang University, Hangzhou 310017, China; 22112222@zju.edu.cn (Z.Z.); jinchenguo0917@163.com (C.J.); 2 School of Civil Engineering, Ningbo Tech University, Ningbo 315100, China; liugenjin@163.com; 3 College of Architectural Engineering, Zhejiang University of Technology, Hangzhou 310018, China; xiazj777@163.com

**Keywords:** 3D printing materials, geopolymers, residue soil, rheology, buildability, mechanical properties

## Abstract

This study investigates the impact of residue soil (RS) powder on the 3D printability of geopolymer composites based on fly ash and ground granulated blast furnace slag. RS is incorporated into the geopolymer mixture, with its inclusion ranging from 0% to 110% of the combined mass of fly ash and finely ground blast furnace slag. Seven groups of geopolymers were designed and tested for their flowability, setting time, rheology, open time, extrudability, shape retention, buildability, and mechanical properties. The results showed that with the increase in RS content, the fluidity of geopolymer mortar decreases, and the setting time increases first and then decreases. The static yield stress, dynamic yield stress, and apparent viscosity of geopolymer mortar increase with the increase in RS content. For an RS content between 10% and 90%, the corresponding fluidity is above 145 mm, and the yield stress is controlled within the range of 2800 Pa, which meets the requirements of extrusion molding. Except for RS-110, geopolymer mortars with other RS contents showed good extrudability and shape retention. The compressive strength of 3D printing samples of geopolymer mortar containing RS has obvious anisotropy.

## 1. Introduction

Three-dimensional printing, also known as additive manufacturing, is defined as “a layered process of fabricating objects by combining materials based on three-dimensional model data” [1]. At present, additive manufacturing is undergoing rapid development in various academic and industrial fields [2,3]. As an advanced construction technique, 3D printing architecture technology can sequentially stack layers through program design and control systems to print objects with complex geometries or entire buildings [4]. Three-dimensional printing technology can be divided into three main categories, including material extrusion, binder jetting, and fused deposition modeling. Among them, material-extrusion-based 3D printing technology, which uses continuous filament extrusion and layer-by-layer forming processes, has the potential for large-scale on-site construction applications [5,6] and is the most widely applied and developed 3D printing technology to date [7,8]. However, material-extrusion-based 3D printing technology imposes stringent requirements on materials, requiring them to be pumpable, extrudable, and have suitable flowability [9,10], and the extruded material must have good shape retention ability [11].

Ordinary Portland cement (OPC) is the most commonly used binder material, and its extraction and production processes are associated with high energy consumption and significant CO_2_ emissions [12,13,14], exacerbating global warming issues. Therefore, in the quest to develop cleaner, greener, and more sustainable 3D printing materials, recent research trends have tended towards the use of low-carbon, green binder materials, in order to reduce cement use and carbon dioxide emissions [15,16]. Compared to OPC, geopolymers have been widely accepted as green binders due to their superior mechanical and durability properties [17,18]. Geopolymers are inorganic polymers produced by the reaction of aluminosilicate materials with alkaline activators [19]. Geopolymers use waste or natural minerals as raw materials, reducing the dependence on natural resources and mitigating negative environmental impacts, thus playing a constructive role in reducing carbon emissions in the construction sector and promoting sustainable development. In recent years, the combination of 3D printing technology with geopolymer has attracted increasing attention [20,21,22].

With the acceleration of urbanization around the world, ongoing infrastructure construction projects are generating large amounts of residue soil (RS). The direct disposal of RS can have adverse environmental impacts and even pose varying degrees of safety hazards. Faced with such a massive amount of RS, it has become the focus of numerous studies [23,24]. The potential for energy saving and emission reduction through the utilization of RS resources is enormous, and the use of 3D printing technology for RS management has promising development prospects. Lou et al. [25] used excavated RS as an additive for chloro-magnesium cement and investigated the influence of RS incorporation on the performance of chloro-magnesium cement 3D printing materials, including mechanical properties, water resistance, setting time, flowability, extrudability, constructability, and curing performance. The results showed that the incorporation of RS improved the mechanical properties and durability of magnesium cement 3D printing materials. Additionally, Mehdi et al. [26] used untreated natural clay and calcined clay as additives incorporated into fly ash–slag-based geopolymer for 3D printing. The results showed that the addition of two differently treated clays significantly improved the printability of the geopolymer, with untreated natural clay performing better in terms of shape stability and improved constructability compared to calcined clay. Ofer Asaf et al. [27] used three types of clays and sands of different particle sizes as raw materials for 3D printing and evaluated the rheological and printing performance of 12 sand–clay geopolymers. This study found that a coarser particle size distribution could increase the static yield strength of geopolymer mortars. Currently, the combination of RS and 3D printing technology is still in the research and development stage, and there is a lack of systematic research on the use of RS as a raw material for the production of geopolymer 3D printing materials. Therefore, this paper investigates the feasibility of using RS for the extrusion-based 3D printing of geopolymer.

The fluidity and setting time of geopolymer mortars are tested, and the rheological properties of geopolymer mortars with different amounts of RS are investigated. In addition, the effects of RS on the extrudability, shape retention ability, and constructability of geopolymer mortars are evaluated. Furthermore, the mechanical anisotropic characteristics are analyzed by measuring the compressive strength of printed specimens in different loading directions after 28 days.

## 2. Raw Materials and Experimental Methods

### 2.1. Raw Materials

**Figure 1:**
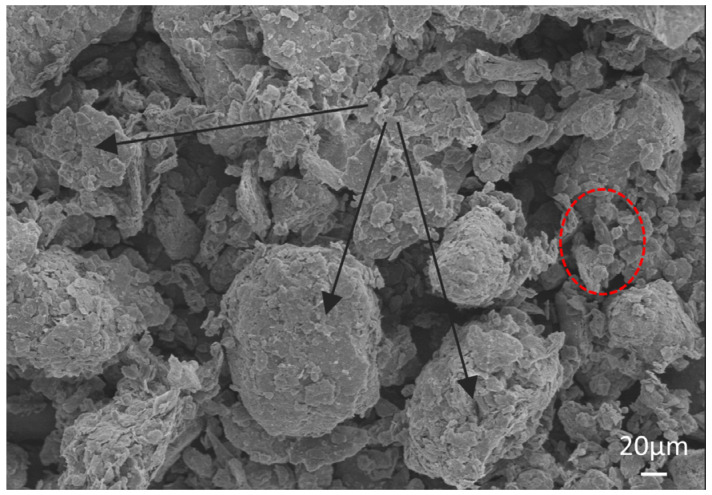
Scanning electron microscope (SEM) of RS.

**Figure 2:**
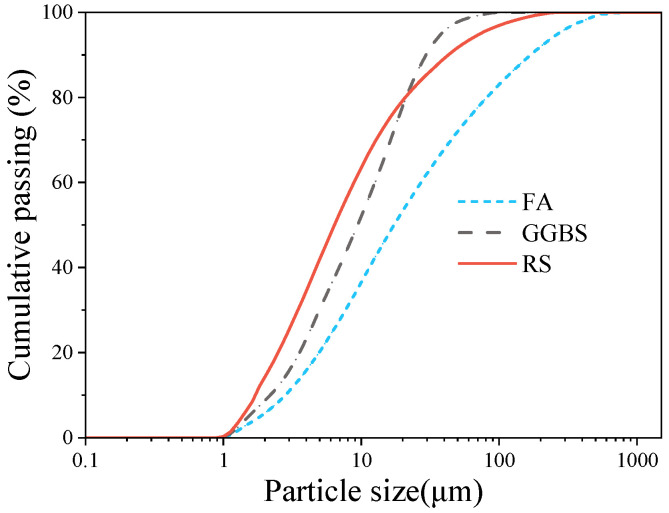
Particle size distribution of the materials used in this study.

Fly ash: Grade II, with a specific surface area of 286.0 m^2^/kg and a D50 of 16.90 µm. Ground granulated blast-furnace slag (GGBS): Grade S95, with a specific surface area of 409.9 m^2^/kg and a D50 of 9.417 µm. The RS was collected from a construction site in Ningbo City with a soil depth of 5 m. After drying and grinding treatment, the RS was sieved to obtain a powder with a particle size of less than 0.15 mm and a specific surface area of 516.7 m^2^/kg, and its D50 was 5.891 µm. The scanning electron microscope (SEM) test results of the RS are shown in Figure 1, showing that the RS has a non-uniform particle size and a high degree of roughness, with angular and uneven characteristics. And the texture shows diversity and complexity. According to the Chinese Standard GB/T 50123-2019 [28], the basic physical properties of the RS were tested, with a plasticity index (Ip) measurement of 18.2. Based on the classification standard of plasticity index, Ip ≥ 17 can be classified as clay. The chemical compositions of FA, GGBS, and RS are shown in Table 1. The particle sizes of the above raw materials were tested using a BT-2002 laser particle size analyzer, and the results are shown in Figure 2. Natural river sand with a fineness modulus of 2.7 was used as the fine aggregate, which is produced in Ningbo. In this study, the admixture used was a polycarboxylate superplasticizer, which is a beige liquid purchased from a supplier in Zhejiang Province. The water-reducing efficiency of this admixture exceeds 25%.

In this study, liquid sodium silicate (Na_2_SiO_3_) with an initial modulus of 3.3 and analytical-grade sodium hydroxide (NaOH, 96%) were used as alkaline activators. The sodium silicate solution and sodium hydroxide were mixed to produce an alkaline activation solution with a modulus of 1.5 and an alkali content of 5%. The prepared alkaline activation solution must be allowed to stand for at least 24 h before use to avoid instability of the experimental results caused by the release of a large amount of heat when NaOH dissolves in water.

### 2.2. Mix Design

The fixed mass ratio of FA to ground granulated blast-furnace slag (GGBS) was 4:1. The RS was incorporated using an external blending method with incorporation levels of 0%, 10%, 30%, 50%, 70%, 90%, and 110% of the total mass of fly ash and ground granulated blast-furnace slag. These are designated as RS-0, RS-10, RS-30, RS-50, RS-70, RS-90, and RS-110, respectively, with RS-0 (0% RS incorporation) as the control group. Lou et al. [25] also adopted a similar approach in their study on the impact of engineering soil on the properties of 3D-printable magnesium cement. Based on past research [29] and the authors’ extensive experiments, it is clear that water content changes have a much smaller effect on the results than RS content changes do. Thus, changes in water content were not separately accounted for in the design of the mix ratio. In this study, solid powders are defined as the sum of fly ash, ground granulated blast-furnace slag, and RS. The water-to-solid mass ratio (i.e., the mass of water to the mass of solid powders) and the sand-to-solid mass ratio were 0.42 and 1.0, respectively, for all samples. After mixing, the modulus of the alkaline activator was 1.5, with an alkali content of 5%, and the dosage of the superplasticizer was 1% of the total mass of fly ash and ground granulated blast-furnace slag. The specific proportions of the mixtures are given in Table 2.

### 2.3. Flowability Test

According to the Chinese standard GB/T 2419-2005 [30], the fluidity of mortar was evaluated to determine the flowability of fresh mortar. A truncated cone mold with an upper internal diameter of 80 mm, a lower internal diameter of 100 mm, and a height of 60 mm was used. The fluidity was determined by measuring the spread diameter of the mortar in two perpendicular directions and taking the average.

### 2.4. Setting Time

According to the Chinese standard GB/T 1346-2011 [31], the initial and final setting times of fresh mortar were tested using a Vicat apparatus. The setting time was recorded from the contact between the alkaline activator and the powder mixture.

### 2.5. Rheological Characteristics

**Figure 3:**
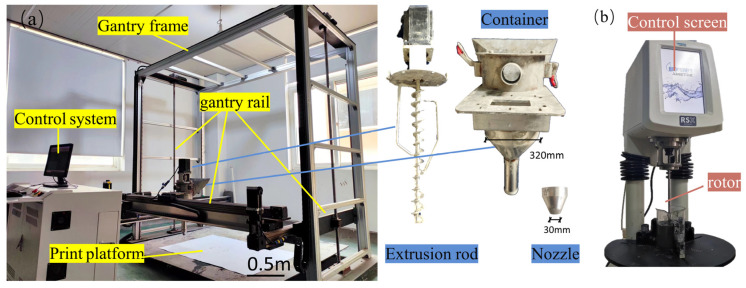
(**a**) Laboratory based on extrusion-type 3D printer; (**b**) rheometer.

**Figure 4:**
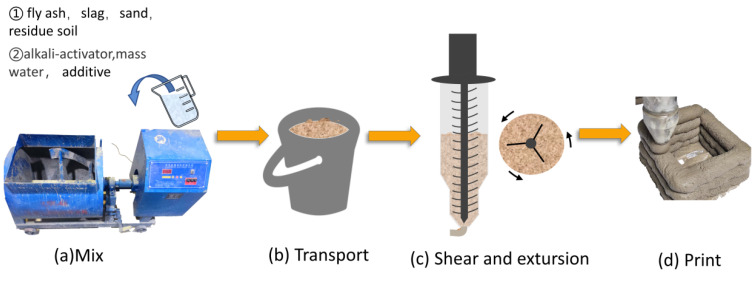
Geopolymer mortar 3D printing process and mortar shear condition.

In this study, a rheometer (as shown in Figure 3b) was used to measure the static yield stress, dynamic yield stress, plastic viscosity, and apparent viscosity of the mortar. This was performed to investigate the rheological effect of RS on geopolymer 3D printing materials and to explore its potential as a 3D printing material. Different shear states were experienced by the mortar during different stages of 3D printing, as shown in Figure 4: (a) mixing stage, (b) stationary stage during mortar transport after mixing, (c) extrusion stage where the mortar is extruded by the screw force, and (d) printing stage where the mortar is stationary again after extrusion. Therefore, it is necessary to simulate the 3D printing process through rheological experiments to understand the deformation and flow characteristics of the mortar in different stages.

**Figure 5:**
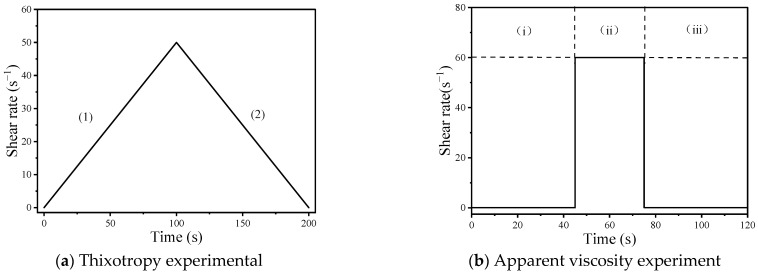
Rheological test scheme.

Higher static yield stress is a necessary requirement for 3D printing materials [32], as it determines whether the material can support the weight of the top structure. Dynamic yield stress refers to the minimum pressure required to pump mortar, while plastic viscosity describes the relationship between external force and flow velocity, with lower plastic viscosity indicating easier flow of the material. The rheological experimental scheme is illustrated in Figure 5a: (1) shear rate is uniformly increased from 0 s^−1^ to 50 s^−1^ within 100 s, during which the shear stress gradually increases to a maximum value representing the static yield stress; (2) shear rate is uniformly decreased from 50 s^−1^ to 0 s^−1^ within 100 s, during which the shear stress gradually decreases with decreasing shear rate. The dynamic yield stress and plastic viscosity can be obtained by fitting the Bingham model equation (Equation (1)) to the experimental data.

(1) $$\tau =\tau_{0}+\mu \gamma$$

where τ is the shear stress, $\tau_{0}$ is the dynamic yield stress obtained after fitting the Bingham model, μ is the plastic viscosity obtained after fitting the Bingham model, and γ is the shear rate.

The apparent viscosity reflects the viscous properties of the mortar flow and indicates the ability of the mortar to resist deformation and flow. If the first layer does not return to its original viscosity before the second layer is applied, structural deformation may occur. The apparent viscosity test scheme for geopolymer mortars was designed to mimic the 3D printing process as shown in Figure 5b. The test scheme consists of three stages: (i) the initial stage, where the material is sheared for 45 s at a rate of 0.1 s^−1^ to simulate the stationary state before printing; (ii) printing state simulation, where the material is sheared for 30 s at a rate of 60 s^−1^ to simulate the state of the mortar being cut and extruded during printing; and (iii) recovery stage, where the shear rate is reduced to 0.1 s^−1^ for 45 s to simulate the stationary state of the mortar after being extruded through the nozzle. By measuring the apparent viscosity in these three stages, the recovery behavior of the geopolymer mortar can be understood, and the entire printing process lasts for 120 s.

### 2.6. Open Time

The open time refers to the ability of the geopolymer mortar to be extruded smoothly through the die without any breakage or uneven phenomena, thus ensuring that the mortar maintains good workability during the printing process. In this study, the open time was measured by extruding a 200 mm strip of mortar with a printing time interval of 5 min.

### 2.7. Printability 

#### 2.7.1. Extrudability

As shown in Figure 3a, extrudability was evaluated using a laboratory extrusion-based 3D printer. The printing parameters were set as follows: the diameter of the nozzle was 30 mm, the speed of movement of the nozzle was set to 50 mm/s, and the speed of rotation of the extrusion bar was 1 r/s. In order to evaluate the influence of the RS on the extrudability of the geopolymer mortar, a six-layer hollow cube with a side length of 160 mm and a width of 32 mm was printed. If the mortar can be extruded continuously and uniformly without any cracks or uneven phenomena, it is considered to have good extrudability.

#### 2.7.2. Shape Retention 

Shape retention is a key indicator for evaluating the deformation and height stability of geopolymer mortars. In this study, a six-layer hollow cube was successfully printed. After 3 h of curing, the height difference (H_d_) between the first layer (H_1_) and the sixth printed layer (H_6_) was measured to investigate the influence of RS on the shape retention of the geopolymer mortar. To ensure the accuracy of the measurement data and the reliability of the results, ImageJ digital image analysis software was used. ImageJ can accurately determine the height of each layer by processing images taken during the printing process.

#### 2.7.3. Constructability

Constructability refers to the ability of 3D-printed products to be stacked layer by layer without warping or collapsing, with the overall size remaining stable over time. In this study, the constructability of geopolymer mortar was evaluated through stacking printing experiments. A hollow cylinder with an outer diameter of 220 mm was printed within the open time. In addition, the extrusion width of each layer of the cylinder was 32 mm and the extrusion height was 24 mm.

### 2.8. Mechanical Properties

**Figure 6:**
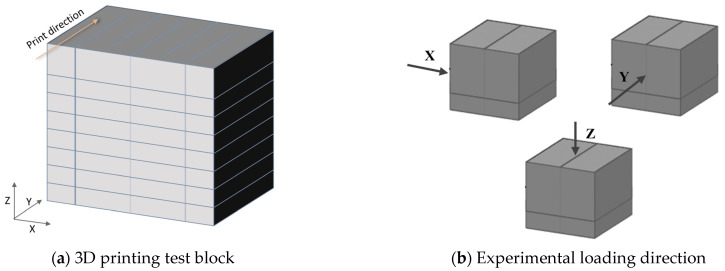
The 28-day anisotropic compressive strength test.

To measure the anisotropic compressive strength of printed samples, solid rectangular prisms measuring 400 mm × 200 mm × 180 mm were first printed, as shown in Figure 6a. Samples measuring 40 mm × 40 mm × 40 mm were then cut from them and cured under standard conditions of 20 ± 2 °C and a relative humidity of no less than 95% for 28 days. The anisotropic compressive strength test used loading directions, as shown in Figure 6b, where “X-direction”, “Y-direction”, and “Z-direction” represent loading directions perpendicular, parallel, and perpendicular to the compression direction, respectively. According to ASTM C109/C109M-13 [33] specifications, three specimens are tested for each direction and the average value is taken as the compressive strength of the specimen.

## 3. Experimental Results and Discussion

### 3.1. Fluidity and Setting Time

**Figure 7:**
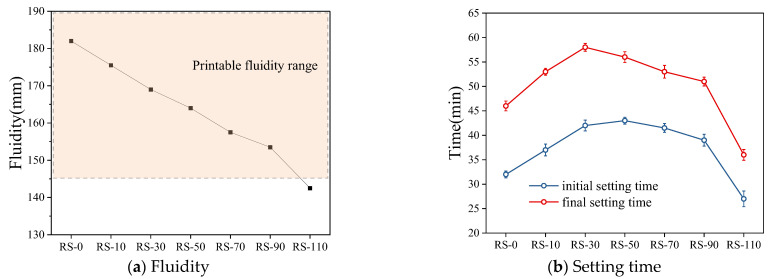
Influence of RS on the fresh mixing performance of geopolymer mortar.

As shown in Figure 7a, the addition of RS has a significant effect on the fluidity of the geopolymer mortar. As the RS content increases, the fluidity of the geopolymer mortar decreases. When the RS content reaches 110% (RS-110), which exceeds 50% of the solid powder content, the fluidity is only 142.5 mm, a decrease of 21.7% compared to the control group RS-0 (182.0 mm), and it no longer meets the extrudability requirements of the mortar. This is because the fine particle size and high specific surface area of the waste particles increase the water absorption rate, absorbing the internal mixing water of the geopolymer, causing the geopolymer mortar to quickly reach a saturated state in a short period, leading to a rapid decrease in fluidity [34]. Although a large amount of water is added simultaneously with the external addition of waste at a fixed water/solid ratio, the fluidity of the geopolymer mortar still decreases significantly as the RS content increases. Therefore, a rise in RS content diminishes geopolymer mortar fluidity, with a notably higher impact than water content. However, too low a fluidity will cause problems such as cracking and unevenness during extrusion, which will severely affect the printing quality and results [35]. Tran et al. [36] suggested that the fluidity of fly ash–slag-based geopolymer should be ≥160 mm to ensure good extrudability. Lou et al. [25] found that MC-3DP slurry has good workability in the range of 186 mm to 213 mm in fluidity. Through a large number of geopolymer mortar 3D printing experiments with waste, it was found that it can ensure good extrudability when the fluidity is >145 mm.

From Figure 7b, it can be seen that as the RS content increases, both the initial and final setting times of the geopolymer mortar show a characteristic of initially increasing and then decreasing. For the RS-0 control group, the initial and final setting times are 32 and 46 min, respectively. When the RS content is increased to 30% (RS-30), the initial and final setting times increase to 42 min and 58 min, respectively, indicating a significant slowdown in the setting rate. This is because the inert components in the waste cannot be activated by the alkaline solution and the waste absorbs a large amount of free water, which is unfavorable for ion migration, resulting in a decrease in the hydration rate of the geopolymer [37,38]. However, for RS-110, the initial and final setting times are only 24 and 36 min, respectively, much lower than for RS-0. This is because the excessively high RS content absorbs a large amount of free water, resulting in severe agglomeration of the geopolymer mortar after mixing, an increase in the initial static yield stress, difficulty in entering the flow state, and rapid attainment of a false setting state.

### 3.2. Rheological Characteristics

**Figure 8:**
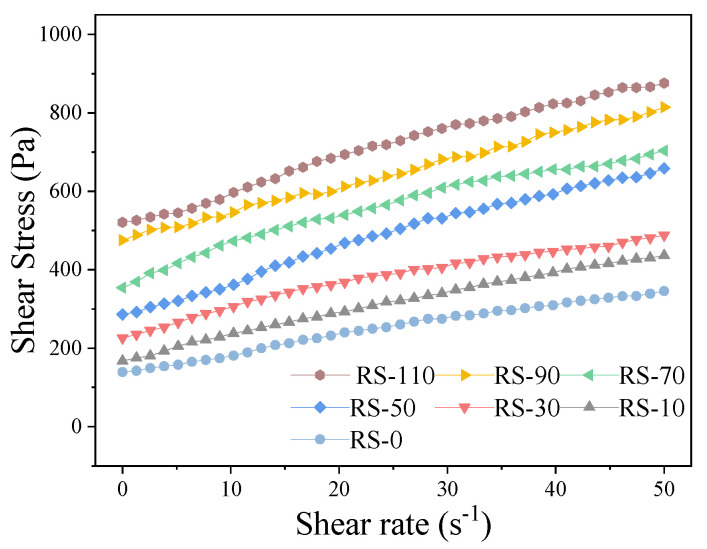
Shear rate-shear stress curve of geopolymer mortar with different RS contents.

The rheological behavior of geopolymer mortars with varying RS content is shown in Figure 8, which shows the relationship between shear stress and shear rate. The results indicate that an increase in RS content leads to an increase in the yield stress of the mortar at the same shear rate. In addition, for all samples, the shear stress increases approximately linearly with shear rate in the range from 0 to 50 s^−1^. Furthermore, for a fixed RS content, the shear stress experienced by the geopolymer mortar gradually increases as the shear rate increases.

The rheological behavior of geopolymer mortars with different RS contents was analyzed using the Bingham model to fit the shear stress–shear rate relationship. The dynamic yield stress and plastic viscosity were then calculated as shown in Table 3. The fitted equations and corresponding rheological parameters have an overall goodness of fit (*R^2^*) greater than 0.99, indicating that the rheological model effectively describes the rheological behavior of geopolymer mortars with varying RS content.

#### 3.2.1. Static and Dynamic Yield Stress

**Figure 9:**
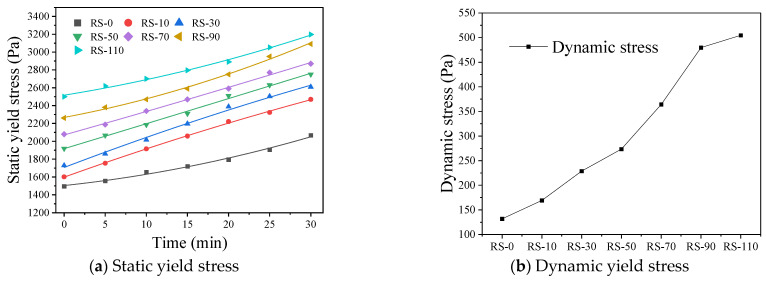
Effect of RS content on yield stress of geopolymer mortar.

Figure 9a illustrates the relationship between the static yield stress of the geopolymer mortar and the RS content. It can be observed that as the RS content increases, the static yield stress of the mortar shows an upward trend. As the RS content increases from 0% to 110%, the static yield stress increases from 1496.4 Pa to 3196.6 Pa, an increase of 113.6% compared to RS-0. This finding is consistent with the results of Dai [39] and Gadkar [40] and indicates that clay particles can increase the yield stress of the material. The reason for this trend is that the high water absorption capacity of the waste powder results in insufficient moisture available in the geopolymer mortar to lubricate the waste particles as the RS content increases. In addition, the thickness of the water film on the surface of the cementitious particles decreases. Thinner water films make the mortarless deformable and flowable. In addition, the irregular shape and rough surface of the waste particles increase the friction between the particles. Therefore, the addition of waste with high water absorption, a rough surface, and a high specific particle surface area significantly increases the static yield stress of geopolymer mortar.

From Figure 9b, it can be seen that increasing the RS content leads to an increase in the dynamic yield stress of the geopolymer mortar, which is in agreement with the findings of Rahul [41] et al. At 0% RS content, the dynamic yield stress of the geopolymer mortar is 131.9 Pa, which increases to 504.6 Pa when the content is increased to 110%, an increase of 282.5%. The increase in the dynamic yield stress makes it more difficult for the mortar to enter the flow state and, consequently, more difficult to pump and extrude. This phenomenon is mainly due to the increased ability of the addition of waste to promote internal aggregation and agglomeration of the geopolymer. Therefore, as the RS content increases, the internal friction and dynamic yield stress of the geopolymer mortar gradually increase.

#### 3.2.2. Apparent and Plastic Viscosity

**Figure 10:**
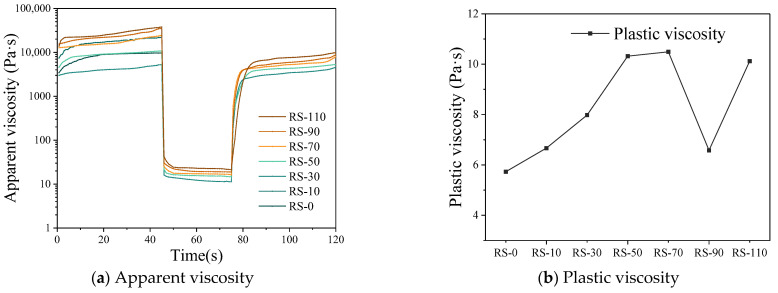
Effect of RS content on viscosity of geopolymer mortar.

Figure 10a shows a comparison of post-shear viscosity at different shear rates for geopolymer mortars with different RS contents. The ability of a geopolymer mortar to recover viscosity after shearing must meet the requirements of pumpability, extrudability, and printability, which are key characteristics for evaluating the printing performance of the material. The results show that as the RS content increases, so does the apparent viscosity of the geopolymer mortar. This is because the van der Waals forces carried by the RS particles lead to agglomeration of the mortar, which is easily broken by the shearing action of the extruder screw. With the addition of RS, the geopolymer mortar will have a higher initial viscosity, in line with the findings of some scientists [20,42]. In addition, after 30 s of high-speed shearing, geopolymer mortar can recover 70–80% of its initial viscosity, and its apparent viscosity gradually increases within 45 s. According to subsequent compression tests, it can support the weight of the second layer without causing deformation of the lower layer.

Figure 10b shows the change in plastic viscosity of geopolymer mortars with different RS contents after shearing. The results show that the plastic viscosity of the mortar varies within a certain range as the RS content increases. At 0% RS content, the maximum plastic viscosity is 5.7 Pa·s, whereas at 70% RS content, the maximum plastic viscosity increases to 10.1 Pa·s, which is 176.6% higher than at 0% RS content. This significant increase in the plastic viscosity of the mortar with increasing RS content is attributed to the increased specific surface area and water demand of the RS, which leads to increased friction in the mortar and therefore an increased resistance to flow, making the mortar more viscous. The increase in plastic viscosity of geopolymer mortars helps to reduce flow deformation, thereby improving the printability of the mortar.

### 3.3. Printability

#### 3.3.1. Open Time

**Figure 11:**
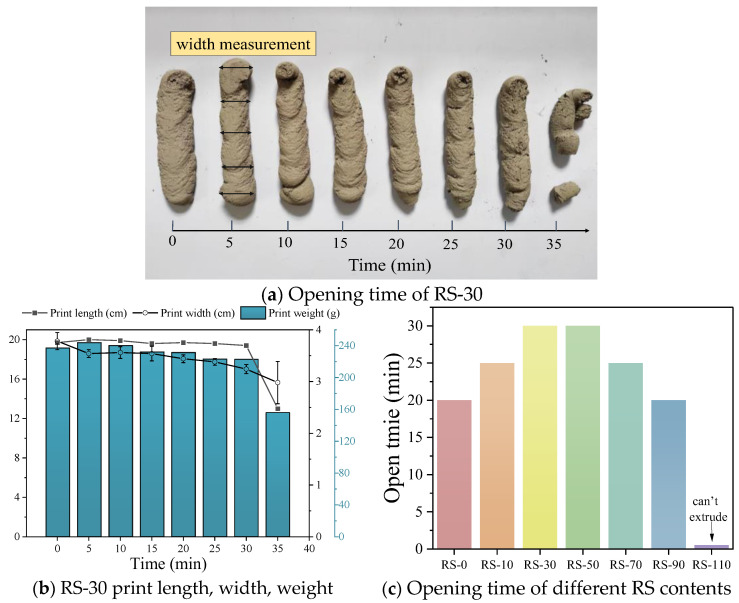
(**a**) Opening time of RS-30; (**b**) printing length, width, and quality of RS-30; (**c**) influence of different RS contents on the opening time of geopolymer mortar.

The open time refers to the time during which the geopolymer mortar can be extruded smoothly after mixing with water. Once this time is exceeded, phenomena such as discontinuous extrusion and breakage occur [18,43]. The quality of extrusion directly affects the accuracy of printing and the quality of printed samples, and extrusion quality changes over time. Therefore, determining the appropriate open time is critical. The open time test results for RS-30 shown in Figure 11a indicate that RS-30 can continuously extrude smoothly within 30 min, but a noticeable discontinuity occurs in the printed mortar bars at 35 min. Therefore, the open time window for RS-30 is 30 min, which is suitable for printing within this time frame. As shown in Figure 11c, except for RS-110, the open time of the other samples is not less than 20 min. Research [18,44,45] has shown that an open time of more than 20 min can better meet the requirements of large-scale 3D printing. In addition, the addition of RS increases the open time of the mortar compared to the control group. This may be because the low reactivity of RS slows down the polymerization reaction rate. The open time is also related to the yield stress of the geopolymer mortar. An increase in RS content leads to an increase in the yield stress of the mortar, resulting in a decrease in open time. Panda [44] used mineral powder instead of fly ash to control the open time and measured the change in yield stress of the geopolymer over time using flow curve tests. The increase in yield stress has a direct effect on the workability of the geopolymer during extrusion, thereby affecting the open time of the geopolymer. Combining the static yield stress results in Figure 9a, good extrudability is achieved when the static yield stress is within 2800 Pa.

When the mortar is fully cured, the length, width, and weight of the extruded mortar must be measured. As shown in Figure 11a, the width of the extruded mortar is measured at five positions, including the head, tail, and middle of the mortar, and the average width is calculated. As can be seen from Figure 11b, the length and width of the geopolymer print change over time, which has a significant effect on the weight of the print. Printability is based on two factors: the ability of the geopolymer to be continuously extruded (i.e., the open window) and the print quality of the extruded mortar (surface smoothness and geometric shape) [46]. Jindal [47] and colleagues quantified the overall quality of printed specimens by calculating the defect area of each specimen. Kazemian et al. [48] found through experimental research that controlling the print width error to within 10% while ensuring dimensional consistency and a continuous extrusion strip is an acceptable print quality standard. In the RS-30 open time test, it can be seen that the print weight changes over time. In terms of extrusion length, there is not much change within 30 min, but at 35 min, there is a discontinuity in the extruded mortar due to the open time being exceeded. Specifically, at the shortest periods, 0 min, 30 min, and 35 min, the extrusion lengths are 197 mm, 194 mm, and 130 mm, respectively. In terms of extrusion width, the widths are relatively stable at 5 min, 10 min, and 15 min, averaging 35.4 mm. However, at 0 min, 30 min, and 35 min, there are significant changes in the extrusion width with widths of 37.2 mm, 32.4 mm, and 25.2 mm, respectively. This phenomenon is due to the fact that when the geopolymer is just mixed, the polymerization reaction starts and the flowability of the mortar is better. Therefore, the compressive strength at 0 min is relatively high. After a certain period of hydration reaction, the extrusion width remains relatively stable within 5–15 min. However, as the geopolymer reaction continues, the static yield stress gradually increases, and when it reaches a certain level, the geopolymer cannot be extruded. Therefore, to accurately control the printability of the geopolymer, several indicators including extrusion length and width should be considered to ensure print quality.

#### 3.3.2. Extrudability

**Figure 12:**
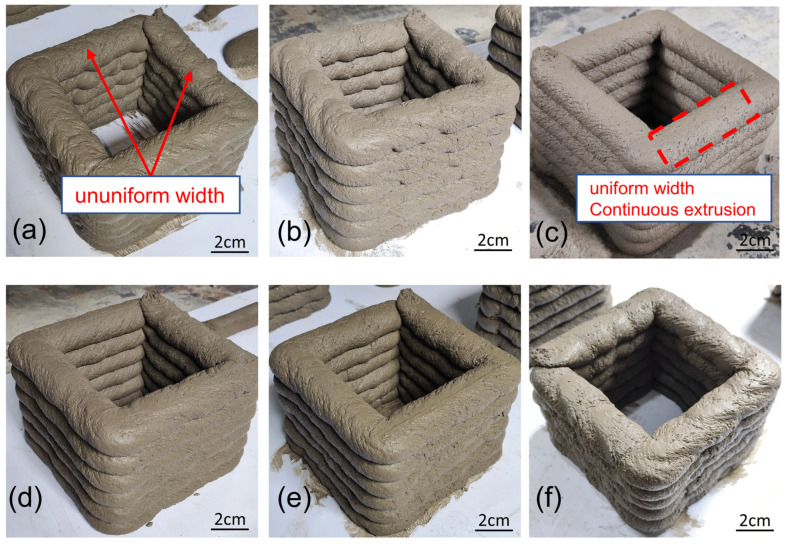
Extrudability of each mix ratio: (**a**) RS-0, (**b**) RS-10, (**c**) RS-30, (**d**) RS-50, (**e**) RS-70, (**f**) RS-90.

As shown in Figure 12, under the experimental conditions of this study, except RS-110, which cannot be extruded smoothly, all other geopolymer mortars can be successfully extruded without clogging the pumping and extrusion systems. RS-110 can smooth the extrusion by increasing the amount of water, but the water demand is very large. This kind of water consumption is not considered in the experimental design of this study. In the future, we will conduct in-depth research and consider increasing water consumption so that high-volume RS can be successfully extruded. Therefore, these mortars have good extrusion performance. Some studies have found that the extrudability of geopolymer mortars can be effectively improved by adding other cementitious materials such as fly ash, silica fume, or additives [49,50]. Guo et al. [49] found that the incorporation of up to 10% silica fume (SF) into FA-based monomeric geopolymer can improve particle filling, effectively increase the viscosity of the geopolymer, and thereby improve the extrudability of geopolymer mortars. Comparing the extrusion situation of RS-30 with the control groups RS-0 and RS-90, it can be observed that the extrusion width is more uniform, indicating that the addition of a quantified amount of RS powder improves the extrusion effect of the mortar. This is because RS contains a large number of clay particles that have volcanic ash activity and pore-filling ability, refining the microstructure of the geopolymer and reducing its porosity [35,47]. Mehdi et al. [26] used natural clay and calcined clay as additives to geopolymer and found that clay can significantly improve the shape stability and constructability of geopolymer. Through maximum compressive load tests with 30 mm of vertical displacement, it was found that natural clay was superior to calcined clay in terms of shape stability at a certain dosage. Consistent with the rheological test results, the appropriate amount of RS increases the static yield stress of geopolymer mortar, which can improve its thixotropic behavior.

#### 3.3.3. Shape Retention Ability

Shape retention ability is a key parameter in 3D printing, directly reflecting the printing quality of geopolymer mortar. During the layer-by-layer printing process, each layer must not only support the weight of the layers above it, but also its weight. Any cross-sectional deformation or collapse can hurt the overall print quality. The shape retention ability of geopolymer grout is assessed by calculating the height difference.

**Figure 13:**
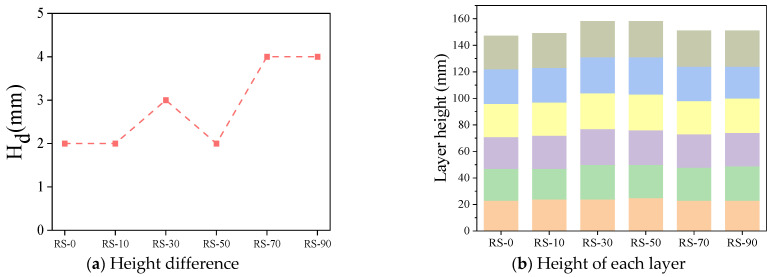
Printed sample: (**a**) height difference, (**b**) height of each layer.

H_d_ is the difference between the height of the first printed layer H_1_ and the height of the last layer H_6_. The smaller the H_d_, the better the shape retention ability. The height retention of all printed samples is shown in Figure 13a, which shows that the H_d_ value increases as the RS content increases. As the RS content increases from 0% to 90%, the cured height differences H_d_ are 2, 2, 3, 2, 4, and 4 mm. This is because as the RS content increases, the flowability of the mortar decreases significantly, resulting in poorer extrudability of the mortar. Figure 13b shows the total height of the printed samples, with RS-30 and RS-50 exhibiting total heights of 160 mm and 158 mm, respectively, an increase of 11.1% and 9.7% compared to RS-0. In addition, RS-70 and RS-90 also have total heights greater than RS-0, indicating that the addition of RS increases the total height of the printed samples. This suggests that RS can improve the shape retention ability of geopolymer mortars, which is consistent with the results of Chougan [26] and Kazemian’s studies [48]. This is because RS particles can absorb more free water, thereby increasing the friction between the solid components of the geopolymer mortar, improving its shape retention ability.

#### 3.3.4. Constructability

**Figure 14:**
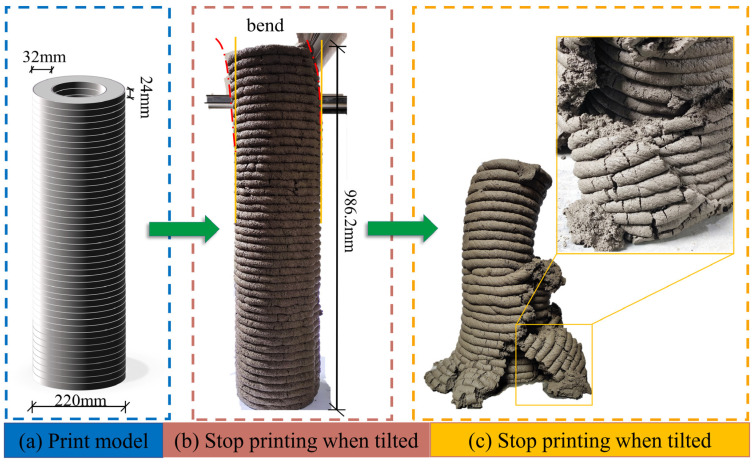
The constructability of RS-30: (**a**) test scheme, (**b**) capsizing state of RS-30 printing 47-layer hollow cylinder, (**c**) details of post-failure state and close-range failure.

The constructability of 3D printing materials is an important property that is a direct reflection of the geopolymer mortar’s ability to maintain shape and resist loads and deformations. Based on the extrudability and shape retention ability of the mortar, RS-30, which exhibited good printing quality, was selected for constructability evaluation. There is currently no standardized test for the constructability of 3D printing. Asaf [27] and Chougan [51] characterized the constructability of 3D printing materials by printing hollow cylinders of different sizes and observing the number of printed layers and printed height, as shown in Figure 14a. From Figure 14b, it can be seen that the RS-30 sample collapsed due to upper lateral bending when the number of printed layers reached 47 layers, with a total height of 986.2 mm at the time of failure. Significant bending of the cylinder was observed throughout the failure process, leading to structural instability. This occurred because the increasing self-weight of the 3D cylinder caused increased lateral bending deformation. At this point, the second-order bending moment at the top of the cylinder also increased continuously. The tensile zone of the cylinder was the first to reach yield strength, eventually leading to buckling failure of the compression member. There were clear signs of imminent failure, with significant crack propagation and noticeable lateral deflection. In addition, as shown in Figure 14c, the cylinder did not completely collapse after failure and the lower-pressure structure still retained good constructability. This is attributed to the material exhibiting a sufficiently high static yield strength. In summary, RS-30 showed good constructability.

### 3.4. Mechanical Properties

**Figure 15:**
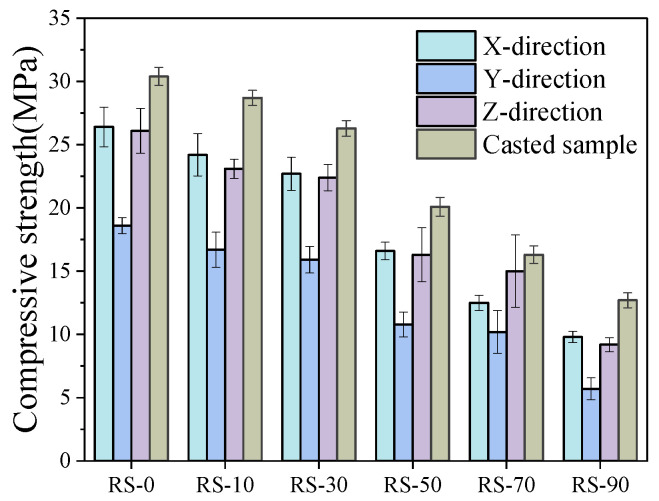
Anisotropic compressive strength of 3D-printed test block after 28 days.

From Figure 15, it can be seen that the compressive strength of the geopolymer mortars decreases as the RS content increases. The compressive strength of RS-10, RS-30, and RS-50 decreased by 6.5%, 13.5%, and 33.8%, respectively, compared to RS-0. In addition, when the RS content exceeded 30%, the compressive strength decreased significantly with increasing RS content. This is because the addition of RS to geopolymer mortar cannot promote the geopolymer reaction, due to its low reactivity. In addition, RS particles become embedded in the gel surface, affecting its density and resulting in reduced compressive strength. Furthermore, the inclusion of RS leads to a higher content of unreacted fly ash particles in the geopolymer [52,53], while excessive RS powder causes excessive self-drying and cracking, which affects the compressive strength of the hardened specimens.

Unlike castings, 3D-printed samples show significant anisotropy due to different loading directions (as shown in Figure 6a,b), with *ƒ*_C_ > *ƒ*_X_ > *ƒ*_Z_ > *ƒ*_Y_. This is consistent with the conclusions of Muthukrishnan [54] and Mechtcherine [55], where the compressive strength is highest in the X-direction. The degree of compaction along the printing direction closely correlates with the printing speed utilized in the experimental procedure; however, this aspect was overlooked by us. The degree of compaction as a function of printing speed is something we need to investigate more thoroughly in the future. Due to the layer-by-layer accumulation during the printing process, the structure becomes denser under the influence of gravity. Consequently, during compression tests, there are fewer cracks formed in the Z-direction of the components. The compressive strength in the X-direction is 76–86% of the cast compressive strength, while in the Z-direction, it ranges from 72% to 92% of the cast components’ compressive strength. However, in the Y-direction, the gaps left between the printed mortar bars can lead to interlayer defects, making it prone to inter-layer defects. These defects can develop into cracks under compression, significantly reducing the compressive strength of the material, with the compressive strength in the Y-direction being 44–62% of the cast compressive strength. In addition, as the RS content increases, the microstructure becomes less dense, resulting in the appearance of numerous large pores. These pores can lead to gaps after pressure hardening, increasing the number of interstitial defects and thus the degree of material anisotropy.

## 4. Conclusions

This study proposes the preparation of geopolymer 3D printing materials containing engineering RS by an external mixing method suitable for extrusion-based 3D printing. Various properties of the 3D-printed geopolymer mortars were investigated and analyzed, including fresh properties, rheological properties, open time, and printability. In addition, the anisotropic compressive strength of printed specimens after 28 days of curing was compared with the compressive strength of cast specimens. Based on the experimental results, the main conclusions are as follows:

1. Increasing the RS content significantly increases the static and dynamic yield strength of the geopolymer mortar, while also increasing the apparent viscosity, which helps to improve printability. Compared to the control group RS-0, the static and dynamic yield strength of RS-110 increased by 113.6% and 282.5%, respectively.
2. Except RS-110, all other materials can be extruded smoothly. A higher RS content considerably lessens geopolymer fluidity, with its detrimental effects greatly outstripping the positives from water content, potentially causing extrusion problems like blockages or uneven flow. For geopolymer 3D printing materials containing RS, good extrudability can be ensured if the flow value is >145 mm.
3. The addition of RS improves the shape retention ability of geopolymer 3D printing materials. However, too much RS can affect the extrudability and reduce the shape retention ability. RS-30 shows good constructability, but increasing structural weight leads to increased lateral bending deformation. The failure occurred when the number of printed layers reached 47 at a printed height of 986.2 mm due to buckling instability.
4. As the RS content increases, the compressive strength of the specimens gradually decreases, and the 28-day compressive strength of the printed specimens is lower than that of the cast specimens in all three loading directions. The compressive strength of geopolymer materials containing RS shows significant anisotropy at 28 days, with the highest value in the X-direction, followed by the Z-direction and the Y-direction.

Our results show that a large amount of RS can be successfully combined with geopolymer materials when the RS is externally mixed, which provides a feasible solution for the reuse of RS. In addition, the incorporation of an appropriate amount of RS improves the printability of geopolymer in 3D printing applications, thereby improving its practical application value.

## Figures and Tables

**Table 1:** Chemical composition of raw materials.

Component	SiO_2_	CaO	Al_2_O_3_	Fe_2_O_3_	MgO	MnO	SO_3_	TiO_2_	K_2_O	Na_2_O
FA	43.3	3.64	44.9	3.27	0.34	0.02	0.80	2.07	0.79	/
GGBS	28.6	41.0	14.30	0.49	9.36	0.32	2.70	1.94	0.42	0.56
RS	57.4	10.7	15.7	4.11	1.84	0.23	0.81	0.92	4.89	2.96

**Table 2:** Mix design.

Mix Group	Soild			Activator (g/1000 g Binder)		Water/Solid Mass Ratio (g/g)	Sand/Solid Mass Ratio (g/g)	Additive (g/1000 g Binder)
	Binder		RS	Na_2_SiO_3_	NaOH			Water Reducer
	FA (in g)	GGBS (in g)						
RS-0	800	200	0	266.1	35.2	0.42	1.0	10
RS-10	800	200	100	266.1	35.2	0.42	1.0	10
RS-30	800	200	300	266.1	35.2	0.42	1.0	10
RS-50	800	200	500	266.1	35.2	0.42	1.0	10
RS-70	800	200	700	266.1	35.2	0.42	1.0	10
RS-90	800	200	900	266.1	35.2	0.42	1.0	10
RS-110	800	200	1100	266.1	35.2	0.42	1.0	10

**Table 3:** Fitting equation and related rheological parameters of geopolymer mortar with different contents of RS, where τ is the shear stress, $\tau_{0}$ is the dynamic yield stress, μ is the plastic viscosity and γ is the shear rate, and *R*^2^ is the coefficient of determination.

Specimen	Fitting Model	$\tau_{0}$ (Pa)	μ (Pa·s)	***R^2^***
RS-0	τ=131.9+5.73γ	131.9	5.73	0.997
RS-10	τ=169.2+6.67γ	169.2	6.67	0.999
RS-30	τ=228.9+7.98γ	228.9	7.98	0.996
RS-50	τ=273.5+10.32γ	273.5	10.32	0.997
RS-70	τ=364.3+10.49γ	364.3	10.49	0.996
RS-90	τ=479.6+8.58γ	479.6	8.58	0.997
RS-110	τ=504.6+10.12γ	504.6	10.12	0.997

## Data Availability

Data are contained within the article.
